# *Bartonella* *quintana* Transmission from Mite to Family with High Socioeconomic Status

**DOI:** 10.3201/eid1801.110186

**Published:** 2012-01

**Authors:** Oto Melter, Mardjan Arvand, Jiří Votýpka, Dagmar Hulínská

**Affiliations:** Charles University, Prague, Czech Republic (O. Melter);; Zentrum für Gesundheitsschutz, Dillenburg, Germany (M. Arvand);; National Institute of Public Health, Prague (J. Votýpka, D. Hulínská)

**Keywords:** *Bartonella* *quintana* infection, mite, transmission, Dermanyssus, Czech Republic, socioeconomic status, vector, bacteria, rash, urban trench fever

**To the Editor:** Urban trench fever caused by *Bartonella quintana* has been reported in persons who abuse alcohol and in homeless persons in large cities worldwide. Symptoms vary from asymptomatic intermittent bacteremia to serious complications (*1*). *Pediculus humanus* lice, the known vector of the infection, are not always identified, which raises the possibility that other vectors might also be involved (*2*). We report on an outbreak of *B. quintana* infection among a young family of high socioeconomic status and their visiting relatives.

The family resides in a regional city (population 104,000) in northern Czech Republic in an old, renovated apartment located on the top floor, just under the roof. In the summer of 2007, hundreds of ectoparasitic mites migrated from a hole in the roof and settled on the inner side of a permanently open window before infesting family members. Two weeks later (day 1 of symptom onset), a papular rash and pruritic vesicular lesions were noted by the parents on the body and legs of their 2 children, a 1-year-old girl and a 3-year-old boy. On day 3, the girl’s body temperature rose to 38.0°C, and the boy’s temperature rose to 39.5°C. The rash resolved in ≈10 days in both children. Vesicular lesions on the girl’s buccal mucosal membrane resolved in 5 days. Excoriated areas resulting from spontaneous rupture of lesions or scratching were still visible on day 14.

On day 4, a fever (temperature, 38.5°C) and intense tibialgia, which persisted for 5 days, developed in the 33-year-old father of the infected children. On day 5, a vesicular rash, which resolved in 10 days, developed in the 33-year-old mother. The children’s grandfather and both grandmothers also showed symptoms of infection within ≈14 days after having spent >1 days or nights in the infected family’s household (Table). In addition, the regional epidemiologist who was involved in the investigation showed development of a severe infection 16 days after exposure to implicated mites that escaped from a collection tube (Table). Recurrent fevers of decreasing intensity, followed by remissions at 1-week intervals, were observed in all patients for up to 3 months.

**Table:** Patient and microbiologic data from a study of *Bartonella* *quintana* transmission from mites to a family with high socioeconomic status, Czech Republic, 2007*

Day after symptom onset†	Date of specimen collection	Specimen type‡	Case-patient	Main symptoms	Specimen testing		Incubation period, d
					IgG titer§	PCR¶	
1	NA	NA	Daughter, son	Papular rash, pruritic lesions	NA	NA	14
3	2007 Jul 5	Serum	Son	Rash, vesicles, fever (temperature 39°C)	Neg	Neg/ND	14
		Serum	Daughter	Rash, vesicles, fever (temperature 39.5°C)	Neg	Neg/ND	14
4	2007 Jul 6	Serum	Father	Recurrent fever (temperature 38.5°C), tibialgia, headache	256	Pos/pos	15
5	2007 Jul 7	Serum	Mother	Vesicles, tibialgia	512	Neg/ND	16
6	2007 Jul 11	Mites	NA	NA	NA	Pos/pos	NA
28	2007 Aug 2	Serum	Epidemiologist	Malaise, arthralgia, headache	256	Neg/ND	16
35	2007 Aug 9	Serum	Grandfather	Malaise, arthralgia, rash, headache	Neg	Neg/ND	14
		Serum	Grandmother 1	Fatigue, malaise	256	Neg/ND	14
		Serum	Grandmother 2	Fatigue, malaise	64	Neg/ND	14
41	2007 Aug 15	Serum	Son	Recurrent fever	256	Neg/ND	14
		Serum	Daughter	Recurrent fever	64	Neg/ND	14
		Serum	Father	Malaise and intense headache	256	Neg/ND	15
		Serum	Mother	Malaise and intense headache	512	Neg/ND	16
		Serum	Grandfather	Recurrent fatigue and malaise	Neg	Neg/ND	14
		Serum	Grandmother 1	Recurrent fatigue and malaise	256	Neg/ND	14
68	2007 Sep 11	Mites	NA	NA	NA	Pos/pos	NA
74	2007 Aug 17	Serum	Epidemiologist	Recurrent fever; fatigue and intense headache	512	Neg/ND	16
163	2007 Dec 13	Serum, B, H	Epidemiologist	Poor concentration, headache	256	Neg/ND	16
197	2008 Jan 17	Serum, B, H	Son	None	Neg	Neg/ND	14
		Serum, B, H	Daughter	None	Neg	Neg/ND	14
		Serum, B, H	Father	Poor concentration, headache	128	Neg/ND	15
		Serum, B, H	Mother	None	128	Neg/ND	16
		Serum, B, H	Grandmother 1	None	Neg	Neg/ND	14

*NA, not applicable; neg, negative; ND, not done; pos, positive; B, blood with anticoagulant EDTA; H, hemoculture. During August 9–19, 2007, children and adult case-patients received oral clarithromycin and oral doxycycline, respectively. On August 9 and 19, 2007, the apartment building in which the case-patients lived was treated with insecticide. †Days after symptom onset do not correlate with incubation period in last column. ‡Specimens were analyzed as follows: serum by serologic testing, EDTA blood by PCR, hemoculture by culture. Patient serum samples were negative for *Anaplasma phagocytophilum* (by immunofluorescence assay [IFA], IgM, and IgG); *Borrelia bugdorferi* (by ELISA and Western blot, IgM, and IgG); *Coxiella burnetii*, *Rickettsia connorii,* and *R. prowazekii* (IFA, total immunoglobulin). §Determined by IFA. ¶Detected by 16S rRNA and by *htrA* amplification.

Seven mites, which were collected by the father on day 6 after symptom onset, were identified as engorged and nonengorged members of the genus *Dermanyssus*. After treatment with ethanol, the mites were investigated by culture and DNA analysis. DNA fragments specific for *Bartonella* spp. (i.e., a 185-bp [*3*] and a 397-bp [*4**,**5*] fragment of the 16S rRNA gene) were amplified; the sequence of the 397-bp fragment was 100% similar to the *htrA* sequence of the *B. quintana* strain Toulouse (Table). Results were negative for PCRs with primers for 16S rDNA of *Anaplasma phagocytophilum* (*6*) and primers for *ospA* of *Borrelia burgdorferi* (*7*). Only *Staphylococcus cohnii* subsp. *urealyticus*, as part of human or animal commensal flora, was detected on blood agar plates that were cultured for 30 days in a microaerophilic atmosphere.

Patient samples were analyzed by using the specific 16S rRNA primers; the *Bartonella*-specific amplicon was found only in a sample that was collected on day 4 from the father. Amplification of the *htrA* gene fragment of identical size and with identical sequences also confirmed the presence of DNA specific for *B. quintana* in the father’s sample. Hemocultures were not performed at symptom onset, but results for patient serum samples cultured under the same conditions as the homogenized parasites remained negative. Significant titers of IgG against *B. quintana* and *B. henselae* or IgG seroconversion in paired serum samples were observed for all patients except the grandfather (Table).

Oral clarithromycin and doxycycline were administered to the children and adults, respectively, for 10 days. The apartment was repeatedly treated with insecticide, and the hole in the roof was repaired, leading to eradication of the mites. The few dead and dry mites that were available for additional parasitologic analysis were mounted in Swan mounting medium (information about the medium is available from the authors), but no characteristics allowing differentiation between species of the genus *Dermanyssus* were recognized during examination by light microscopy. Failed attempts were made to trap pigeons that had lived on the roof of the apartment or in the same city; however, samples from trapped synanthropic pigeons from the north (n = 20) and central (n = 33) part of the country were negative for *Bartonella* spp. by the culture and amplification methods described above. Recurrent fever reported by adult patients resolved in 3 months, and all patients made a full clinical recovery. Laboratory findings for the patients were followed for 6 months after symptom onset (Table).

The fact that the suspected vector was a hematophagous mite (*Dermanyssus* sp.), a parasite of synanthropic pigeons and a suspected vector of other bacterial pathogens (*8**,**9*), and that the 16S rRNA *Bartonella* spp. gene was detected in mites (*Steatonyssus* sp. from the superfamily *Dermanyssoidea*) (*10*) remains a challenge for additional study. Pigeons probably played the role of accidental host in this outbreak, but the source of the infection remains unclear.
